# Initial experience of an investigational 3T MR scanner designed for use on neonatal wards

**DOI:** 10.1007/s00330-018-5357-7

**Published:** 2018-04-30

**Authors:** Paul D. Griffiths, Deborah Jarvis, Leanne Armstrong, Daniel J. A. Connolly, Pauline Bayliss, Julie Cook, Anthony R. Hart, Elizabeth Pilling, Tamanna Williams, Martyn N. J. Paley

**Affiliations:** 1 Academic Unit of Radiology, University of Sheffield, Floor C Royal Hallamshire Hospital Glossop Road, Sheffield, S10 2JF UK; 2 Sheffield Teaching Hospitals Foundation Trust, Sheffield, UK; 3 Sheffield Children’s Hospitals Foundation Trust, Sheffield, UK

**Keywords:** Magnetic resonance imaging, Magnetic resonance spectroscopy, Magnetic resonance angiography, Neonate, Brain

## Abstract

**Objectives:**

MR imaging of neonates is difficult for many reasons and a major factor is safe transport to the MR facilities. In this article we describe the use of a small, investigational 3-T MR customised for brain imaging and sited on a neonatal unit of a tertiary centre in the UK, which is in contrast to a 300-m journey to the whole-body MR scanner used at present for clinical cases.

**Methods:**

We describe our methods for preparing babies for safe transport and scanning on an investigational 3-T MR scanner on a neonatal unit and the development of appropriate MR sequences. The MR scanner does not have CE marking at present so this early development work was undertaken on normal neonates whose parents consented to a research examination.

**Results:**

Fifty-two babies were scanned and there were no serious adverse events. The MR examinations were considered to be diagnostically evaluable in all 52 cases and in 90% the imaging was considered to be at least as good as the quality obtained on the 1.5-T scanner currently used for clinical cases.

**Conclusion:**

We have shown that this investigational 3-T MR scanner can be used safely on a neonatal unit and we have refined the MR sequences to a point that they are clinically usable.

**Key Points:**

*• Access to neonatal MR imaging is limited.*

*• We describe an investigational 3-T MR scanner site on a neonatal unit.*

*• The scanner produces images suitable for clinical practice.*

## Introduction

Magnetic resonance (MR) imaging has revolutionised the assessment of the brain in clinical practice and is the most accurate radiological method for detecting most brain pathologies in most age groups. The ability of MR imaging to produce high spatial and contrast resolution images without exposure to ionising radiation has ensured rapid clinical uptake. One group that has not benefited from MR imaging as much as others is neonates, in whom MR imaging of the brain is difficult. There is inherently poor contrast resolution in the neonatal brain due to its immature state of myelination and obtaining high-quality MR images in a non-sedated/non-anaesthetised baby is challenging because of movement.

A further problem is ensuring safe transfer of the baby from the neonatal units to the MR scanner. Many neonates who may benefit from MR imaging of their brain have unstable cardiovascular and respiratory function and transfer to the MR scanners (usually in a different part of the hospital or at a different hospital) introduces extra risk. The decision to perform a clinical procedure, including a diagnostic test, should be considered on the basis of a risk/benefit analysis and, for neonates, the decision is often not to perform MR imaging. In such cases, transfontanelle cranial ultrasound scanography (cUSS) is often relied upon as it can be done on the neonatal wards and has been used successfully over many years. These factors are well illustrated in a short educational video made at Sheffield Teaching Hospital by the Wellcome Trust [1].

It has been a long-term aim of our group to improve access to MR imaging of the brain in neonates and in this article we describe our initial experience of using a physically small, but high field MR scanner installed in the neonatal unit.

## Methods

**Fig. 1:**
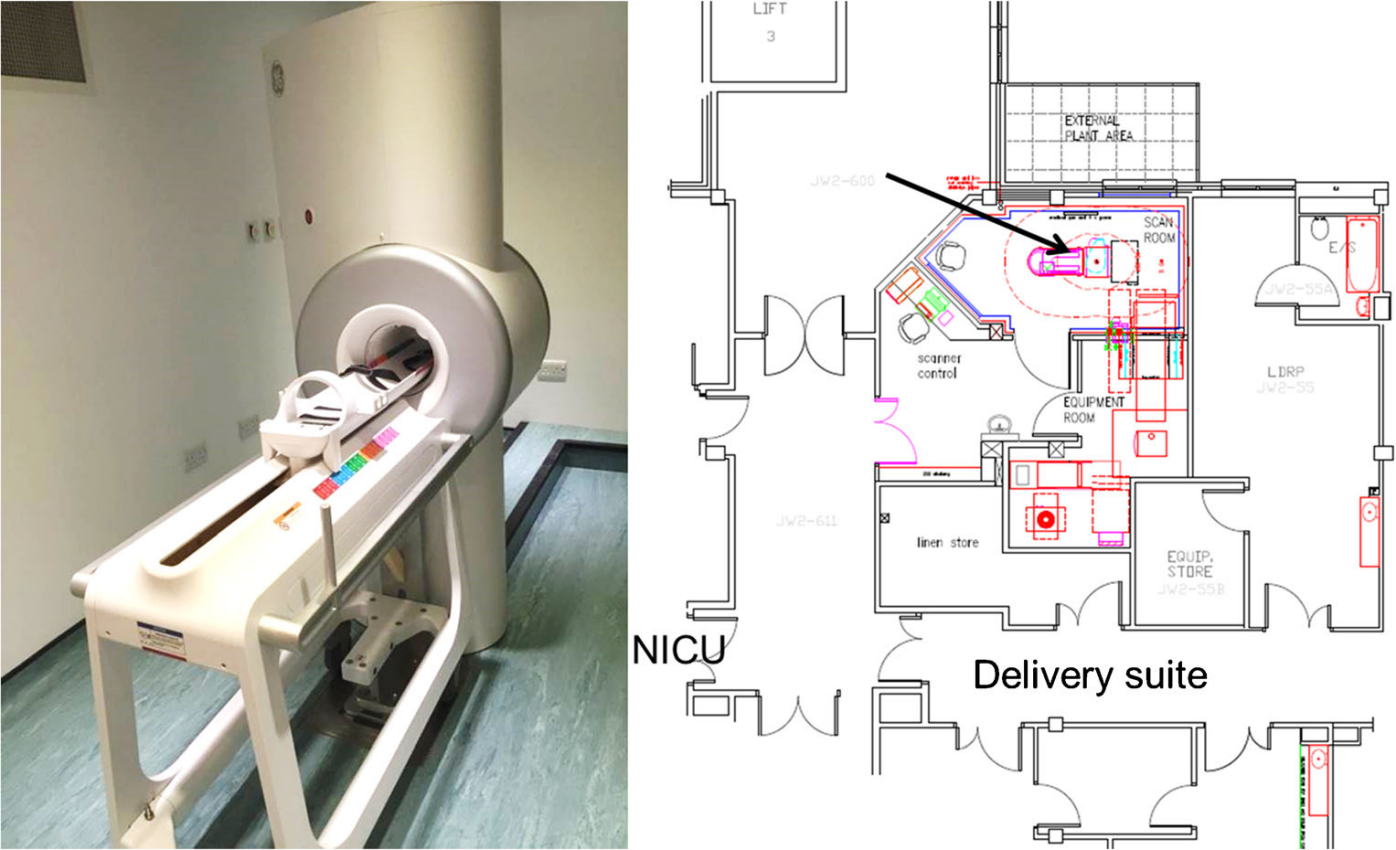
The GE Healthcare investigational 3-T neonatal MR system installed at Sheffield Teaching Hospital and a plan of the MR suite showing the proximity to the neonatal intensive care unit and delivery suite. The scanner control, scan room and equipment room occupy a floor space of 6.0 m × 6.5 m

The investigational high-field (3-T) neonatal MR scanner MR employed in this study was designed and built by GE Healthcare, Milwaukee, WI, USA, and can be sited on non-reinforced office floors (weight 500 kg) in an area of approximately 6 m^2^ including scan control and equipment rooms (Fig. 1). It is designed to have the full capability of a current clinical MR system but customised to scan neonatal brains. It was installed on the Neonatal Unit of our institution resulting in much shorter journeys for neonates to have a MR scan in comparison with the 300 m (including two lift journeys) to the existing MR facility in the radiology department. The 3-T static magnet is a closed loop cryogenic system with a low helium volume (30 l), which maintains the magnet at 4.2 K. The 5-Gauss fringe field is contained within the scanner room (2.0 m × 1.3 m) and has a field uniformity of 15 ppm within a 15-cm-diameter sphere at the iso-centre. The maximum gradient strength is 70 mT/m and the maximum slew rate is 300 T/m/s. dB/dt is kept below the FDA guidelines of 300 mT/m/ms by software and hardware control. The RF power of the transmitter is set at 4 kW to allow maximum SAR levels in the range of 2W/kg for babies weighing up to 6 kg. The magnet bore has 28.0 cm diameter and 50 cm length, the gradient bore is 21.8 cm and the internal diameter of the quadrature transmit-receive RF coil is 17.9 cm, which is the only coil available at present. The magnet, gradients and RF systems are linked to a clinical ‘front-end’ running software at release level DV25.0.

### Ethical approval

The investigational MR scanner currently has neither US Food and Drug Administration (US FDA) approval nor European Conformity (CE) marking, so its use was governed by the Medicines and Healthcare products Regulatory Agency (MHRA) of the UK. The MHRA specified that the system could not be used for primary diagnostic purposes and the manufacturer should be the sponsor of the study. Ethics approval for enrolment of up to 60 subjects was granted by a specialist paediatric Research Ethics Committee, which allowed the researchers to approach the families of two groups:

- Group 1.Normal babies born at term (≥ 37 weeks gestational age, GA) whose parents agreed to have an MR study before the baby was discharged
- Group 2.Premature babies that were about to go home at term-corrected age and had had clinical neuroimaging studies (cUSS) performed at an earlier date

All parents provided written, informed consent.

### Safety issues and patient handling

The pre-checks and preparation for the MR scan were carried out by one of two specialist research nurses associated with the study (PB, JC). The scans were scheduled to be within 1 h of the baby being fed. We confirmed that the baby’s head would fit into the scanner by using a soft template with the same internal diameter as the scanner before leaving the ward. None of the recruited babies were excluded on the basis of head size and there were no scan failures due to head size. There are no specific safety concerns about exposing a neonate to a 3-T static magnetic field [2], although the strong field gradient at the entry to this magnet (39.8 T/m) requires a strict protocol to prevent ferromagnetic materials from entering the scan room. Parents completed MR safety screening forms for their baby and themselves, as one parent was allowed to accompany their baby during scanning. Any monitoring device or other equipment not compatible with MR scanning was removed, and the baby was changed into a Velcro-fastened vest. Screening for metal was completed on arrival at the MR unit by the radiographer (DJ) who ensured all ferromagnetic personal items were removed from the adults and visual inspections of the baby were supplemented by the use of a hand-held metal detector.

Body temperature was first measured on the ward using a hand-held electronic thermometer and on arrival at the scanner control room. A disposable temperature probe was attached to the baby’s axilla and once the baby was on the scanner the probe was connected to an MR-compatible monitoring system (Invivo Expression MR400, Philips Healthcare, Eindhoven, The Netherlands). Temperature was monitored continuously during scanning, along with heart rate and the oxygen saturations of the baby. The temperature was recorded on return to the ward. A further issue is the acoustic noise created by the scanner, which can have equivalent continuous sound levels of around 102 db (A) for some of the planned MR sequences. As such, noise reduction of at least 22 dB was required to comply with the IEC 60601-2-33 standards and was achieved by a combination of earplugs and ear protectors. Mack’s® mouldable earplugs (McKeon Products, Inc., Warren, MI) gave a noise reduction rating of 22 dB (manufacturer’s data) and the additional use of MiniMuffs® (Natus Medical Inc. Pleasanton, CA) provided an extra 7-dB noise reduction (manufacturer’s data).

**Fig. 2:**
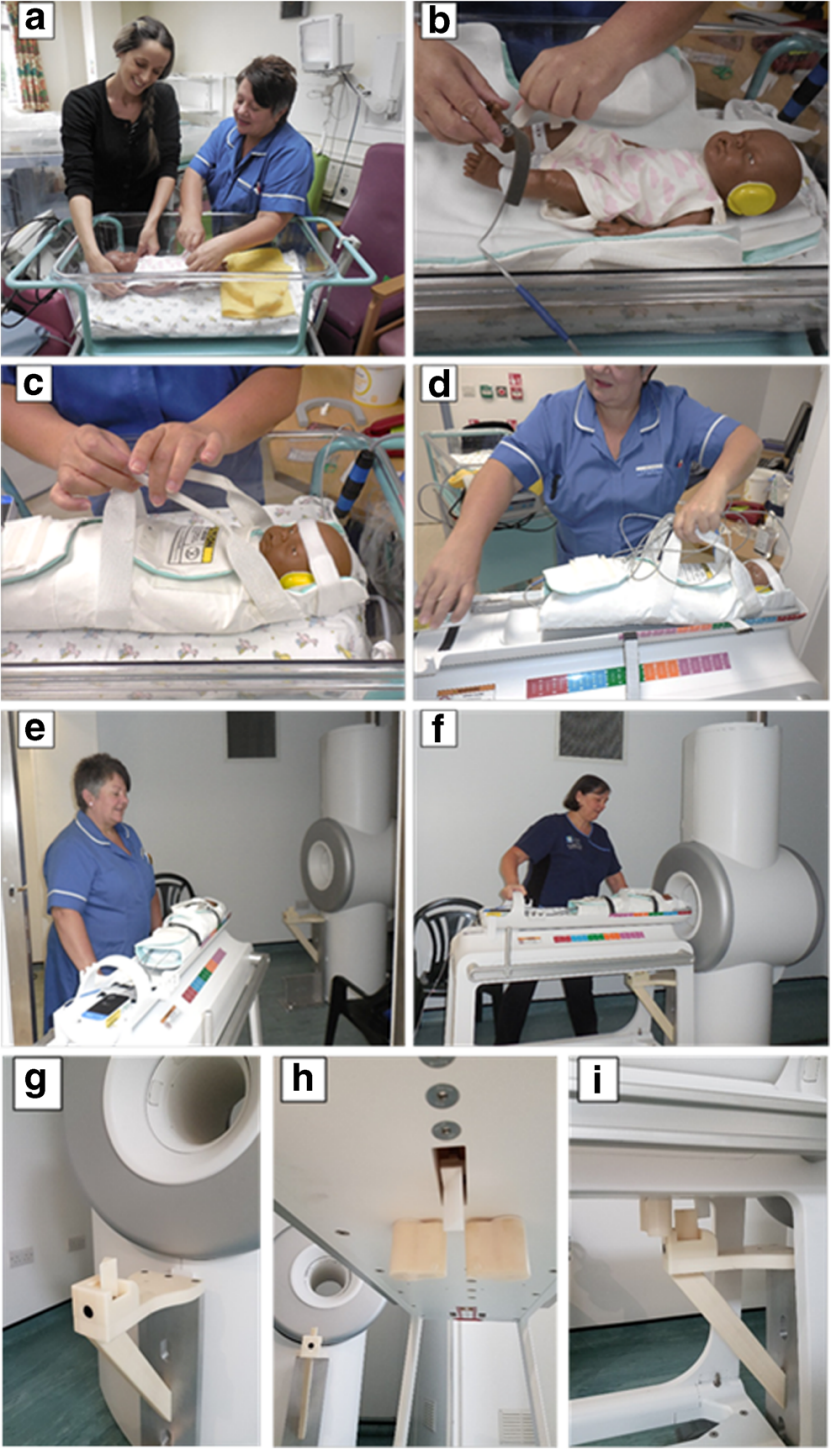
Preparation of a baby for MR scanning on the GE Healthcare investigational 3-T neonatal MR scanner illustrated by a ‘mock’ case using a mannequin and research staff (see text for full details). The baby is changed into clothes with no ferro-magnetic components on the ward and an axillary temperature probe is attached (**a**). After transfer to the atrium in the MR suite the baby is re-checked for absence of ferro-magnetic items and earplugs, ear defenders and a vital sign monitor are applied (**b**). The baby is placed in a transport sling (**c**) and transferred on to the scan cradle of the tabletop, which has been detached from the MR scanner (**d**). The table and baby are pushed into the scanner room (**e**), the table is docked with a scanner and the cradle manually slid into the bore of the MR scanner (**f**). Close-up images of the scanner/table docking mechanism are shown in **g** to **i**

Babies were transferred from the ward to the MR suite in their standard-care cot by the specialist research nurse accompanied by the parent(s) (Fig. 2). The baby was placed in a disposable sling, which swaddled them closely to minimise motion, help maintain body temperature and provide a secure method of transfer into the cradle of the scanner. The scan cradle is built into the scan table, which is detached from the scanner and brought into the control room. The scan table holding the baby was then taken into the scan room and manually docked with the scanner. A slide system with dual rulers on the bed and scan cradle were used to position the baby’s head at the iso-centre of the scanner without the need for electrical drives for the table or positioning lasers. In the event of an emergency or subject distress, the bed can be rapidly detached from the scanner and brought into the scan control room, where appropriate care can be provided.

### MR methods

Exposure to the magnetic field was limited to 1 h, including time allowed for acclimatisation to the scan room, monitoring at the start and end of scanning, and positioning of the baby in the scanner. The research nurse stayed with the baby during scanning for visual monitoring and to stop the scan if there was any concern. Approximately 40 min of image acquisition time was available during an uneventful scan event. We wanted to optimise a full range of imaging sequences (including MR angiography) on the investigational scanner to mirror our current clinical MR imaging protocol performed on a 1.5-T scanner (Table 1). Development of the MR protocol involved refining and trialling a single sequence before focusing on the next sequence. To begin with the imaging parameters for the T2-weighted imaging were modified until the optimal contrast, resolution and scan time were achieved for both ultrafast single shot fast spin echo and T2 FSE imaging. Those optimised sequences were acquired in all subsequent cases and long TE single voxel proton spectroscopy and T1-volume imaging sequences were added and trialled in cases 6-11. Diffusion-weighted imaging and susceptibility-weighted imaging (both gradient echo T2* and sensitivity-weighted imaging) were added in cases 9-13. MR arteriography and venography sequences were evaluated and refined during the scans on cases 13-18. All sequences were acquired in cases 20-49 with further fine-tunings to ensure full optimisation. The last five cases were scanned with a protocol provided by the sponsor of the research study to provide the data suitable for application for CE marking.

**Table 1:** Summary of optimised MR sequence data used in the present study

								Sequences for vascular imaging	
	2D T2 ssFSE	3D FSPGR T1 Volume	2D Propeller DWI	2D GE	2D T2 FSE	3D SWAN (susceptibility-weighted imaging)	Single-voxel spectroscopy	3D MRA (time of flight)	3D MRV (phase contrast)
Repetition time (TR)	908 (min)	10	6262	600	7000	57.5	1500	21	35
Time to echo (TE)	140	Minimum full	60	15	124	23	144	3.4	5
Flip angle	90	8	110	15	142	25	90	20	10
Bandwidth (KHz)	31.25	15.6	50	15.6	15.6	31.3		27.8	21
Prep time	-	900	-	-	-	-	-	-	-
Echo train length		-	20	-	16	5	-	-	-
NEX (signal averages)	0.55	0.75	2	2	2	0.7		1	0.63
Slice thickness/slice gap (mm)	3/0	1.0/0	4/0	3.0/0.3	3/0	3/0	15	1/0	2.4/0
Field of view (cm)	16 × 14.4	18 × 12.6	16 × 16	16 × 12	16 × 14.4	16 × 11.2	1.5 × 1.5	20 × 14	20 × 13
Freq/phase matrix	256/192	256/192	128/128	156/192	256/192	256/192		384/224	320/192
Interpolated matrix and slice thickness		512/384/0.5			512/384/1.5	256/384/1.5		384/224/0.5	320/384/1.2
b Value	-	-	1000	-	-	-	-	-	-
Scan time (s)	29	264	192	180	231	173	140	240	364
Additional					Flow comp.	Flow comp.		Flow comp.	VENQ = 10

The images from each baby were reviewed immediately after the study by one or both of the two paediatric neuroradiologists involved in the study (DJAC, PDG) to report any unexpected intracranial findings to the neonatal staff. Two to three weeks later the images were formally reviewed for the purpose of the study by both reporters by consensus to assess the quality of the imaging and recommend any changes to the sequence parameters for future scanning. The neuroradiology reporters were asked to rate the overall quality of the imaging data set as either (1) fully evaluable (each sequence is of diagnostic quality), (2) partially evaluable (some sequences degraded but a clinical report would have been possible) or (3) not evaluable, i.e. a clinical report could not have been made. The assessors were also asked to compare a number of aspects of image quality against the current reference standard at our institution, namely MR imaging at 1.5 T. ‘Poor’ was used if the quality measures were worse than the current clinical standard, ‘average’ if comparable and ‘good’ if better than current 1.5-T images.

## Results

Fifty-four subjects were consented but in one case consent was withdrawn before the MR study was performed, and in another case, MR scanning was attempted but no diagnostic images were obtained because of equipment failure. Of the 52 babies with successful MR imaging, 60% were born at term and approximately 40% were born before 37 weeks GA. The median corrected gestational age at the time of the MR study was 39 weeks (interquartile range 36-40, full range 34-43 weeks GA), median weight was 2.8 kg (interquartile range 2.4-3.4 kg, full range 1.3-4.5 kg) and median head circumference 34.0 cm (interquartile range 32.6-35.8 cm, full range 28.3-39.0 cm). There was one reportable adverse event—a term baby with a pre-existing baseline bradycardia was recognised as having bradycardia during scanning. A 12-lead ECG study had confirmed normal sinus rhythm previously and the bradycardia was judged not to be attributable to the MR scan so the baby was discharged on the day of the scan and did not meet the local criteria for follow-up.

**Fig. 3:**
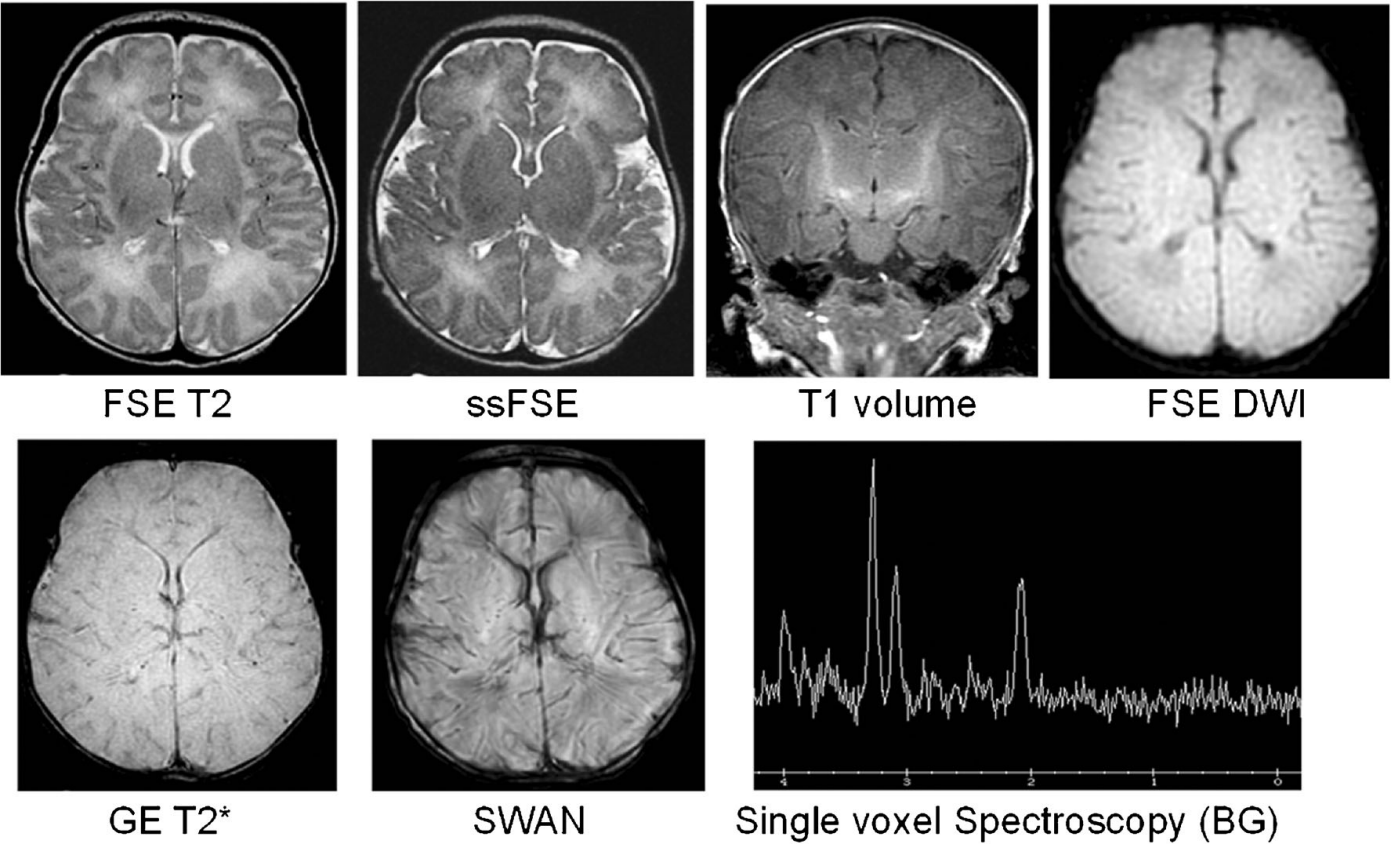
Representative MR images from a normal baby born at term showing the range of routine sequences acquired in cases 20-49 in this study

**Fig. 4:**
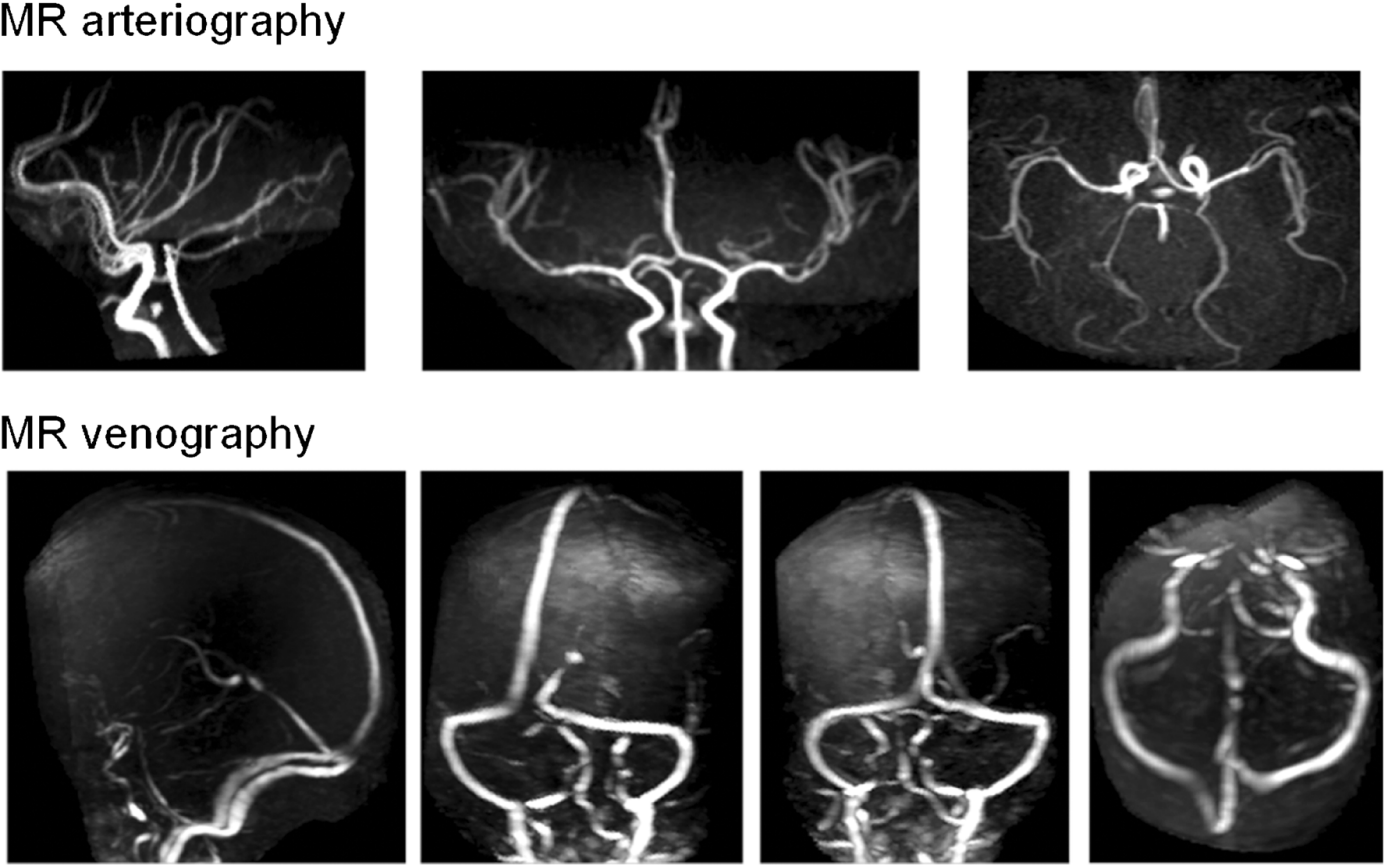
Representative images from a normal baby born at term showing MR arteriography and MR venography image sequences optimised in cases 13-18 in this study

The MR imaging studies were considered to be evaluable in 52/52 (100%) subjects, of which 22/52 (42%) were fully evaluable and 30/52 (58%) were partially evaluable (Figs. 3 and 4). The image quality was judged to be at least comparable to the clinical 1.5-T scanner in 47/52 (90%) of cases and better in 34/52 (65%). Similar results were found for image contrast, signal-to-noise ratio, tissue contrast and homogeneity. The worst results were for artefacts, which were thought to be worse than expected at 1.5 T in 13/52 (25%) of cases (Table 2) and were predominantly due to subject movement.

**Table 2:** Imaging quality assessments made by the paediatric neuroradiology experts

	Poor	Average	Good	Percentage of ‘average’ or ‘good’ cases
Overall image quality	5	13	34	90%
Image contrast	4	8	40	92%
Presence of artefacts	13	20	19	75%
Signal-to-noise ratio (SNR)	7	7	38	87%
Tissue contrast	2	9	41	96%
B_0_ inhomogeneity leading to fat/water separation	0	22	30	100%

**Fig. 5:**
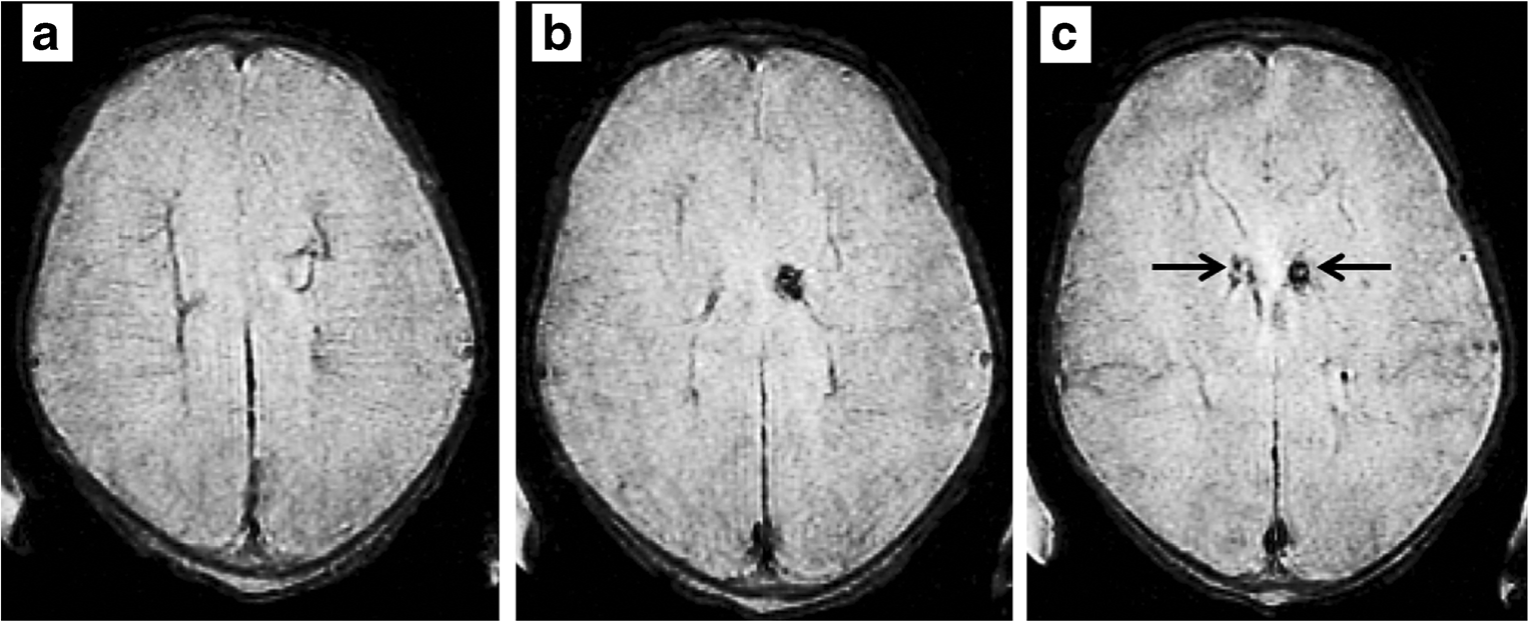
Representative axial gradient echo T2* images from case A described in the text showing small areas of haemosiderin staining in the caudate-thalamic notches bilaterally consistent with previous haemorrhage into the remnants of the germinal matrix (arrowed, **c**)

**Fig. 6:**
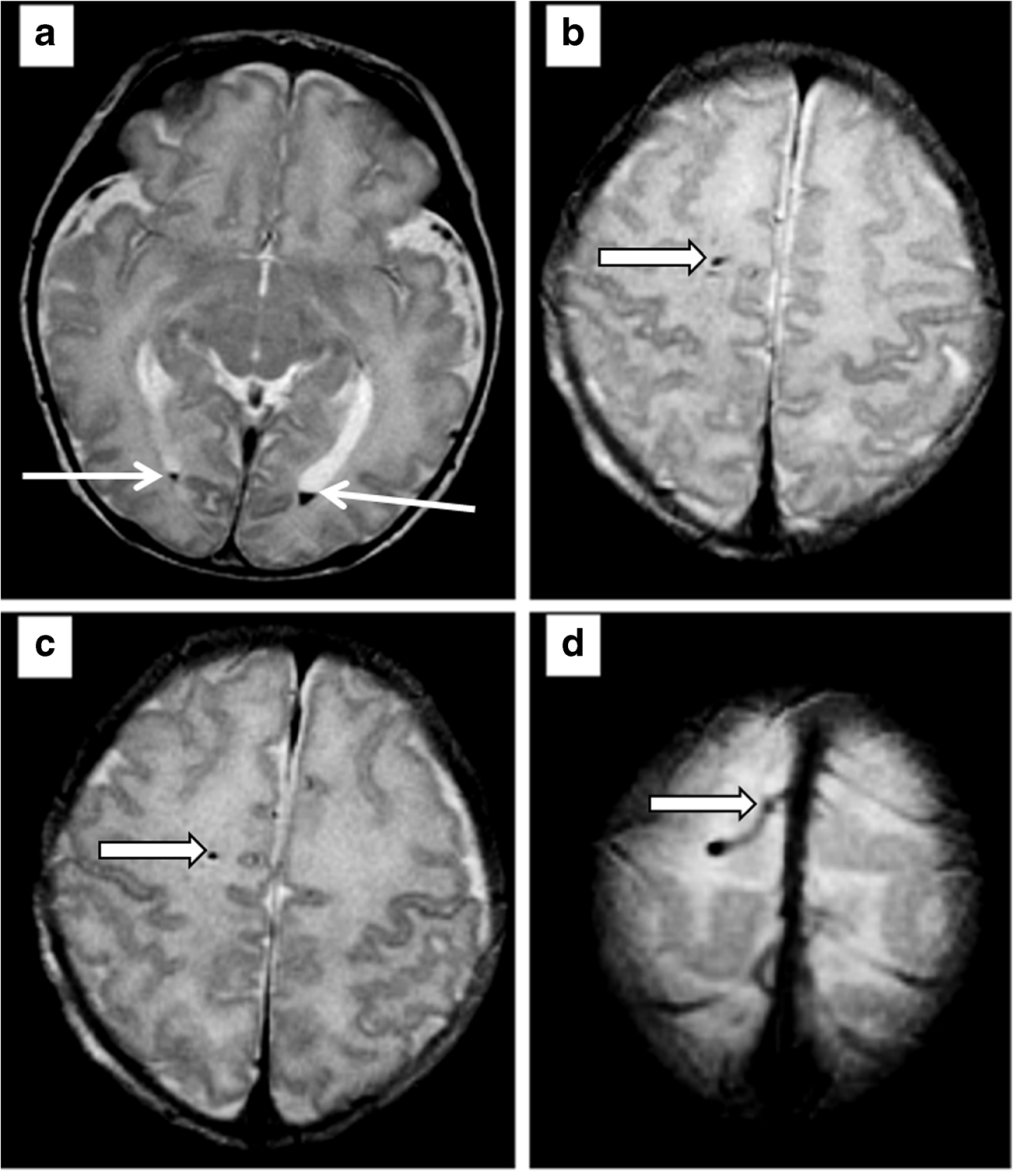
Representative axial FSE T2* images from case B described in the text showing a small volume of intraventricular blood in the gravity dependant parts of the occipital horns of the lateral ventricles (arrowed, **a**) and a tubular structure running through the right frontal lobe and terminating in the superior sagittal sinus (block arrows, **b**-**d**) in keeping with a developmental venous anomaly

Two babies had unexpected intracranial findings on their MR examinations that required input from the neonatology staff:

- **Case A** (Fig. 5)**.** A boy born at 37 weeks GA and scanned on day 1. Haemosiderin staining of the ependyma was shown in both occipital horns and caudate-thalamic notches consistent with earlier haemorrhage. Clinical review the following day was unremarkable and the baby was allowed home with no planned follow-up.
- **Case B** (Fig. 6). A boy born at 37 weeks GA was scanned on day 2. There was a small intra-ventricular haemorrhage and a developmental venous anomaly in the right frontal lobe. Clinical review the following day was unremarkable and the baby was discharged. He was reviewed in clinic at 2 months and was developing normally.

## Discussion

We have demonstrated that a physically small, 3.0-T MR scanner is a feasible option for imaging the neonatal brain on a neonatal unit without the need for additional major structural alterations. There are other methods for improving access to neonatal MR imaging such as MR-compatible transport incubators, which reduces the number of times the baby has to be handled during transfer and provides a controlled physical environment during scanning. The MR compatible transport incubator has a built-in MR coil allowing the baby to go into the scanner while in the incubator thereby reducing the number of handling events. Our group was involved in the early prototype trials of one such transport incubator system [3] and we have designed and built our own MR-compatible incubator [4], which is used clinically.

We maintain that an MR scanner on the neonatal unit is a fundamentally superior approach. It is generally not possible to install whole-body MR scanners on the neonatal unit in most hospitals because of the space required and floor loading. Hospitals with a whole-body MR scanner installed in the NICU have usually done so by designing a new department. Other groups have approached the problem by developing MR services optimised to meet the needs of imaging neonates on standard clinical MR scanners (1.5 T and 3 T) by using tailored equipment and/or care measures [5, 6]. There have also been other attempts to construct small footprint systems for neonatal imaging. Our group previously reported experience of using a 0.2-T permanent magnet system for neonatal imaging [7]. More recent developments have used a 0.023 T system [8] and the US FDA has recently cleared a small, 1.0-T neonatal MR system for neonatal use [9]. We came to the conclusion, based on our experience of neonatal imaging at 0.2 T, that the detailed requirements for neonatal brain imaging (e.g. spectroscopy, diffusion imaging, MR angiography) include at least 1.5 T. A similar view was reached by Tkach who successfully developed a small-footprint 1.5-T system sited on a neonatal unit that can image several anatomical areas [10]. They have subsequently reported their experience of imaging 492 premature neonates [11]. We believe that the theoretical improvements in the signal-to-noise ratio provided by imaging at 3 T offer the best potential imaging for the neonatal brain because of the requirements of high-quality multi-sequence imaging/spectroscopy in as short a time as possible. It should be noted that the US FDA guidelines confirm that MR devices with main static magnetic fields of ≤ 4.0 T should be classified as a ‘non-significant risk’ for neonates [12]. There are disadvantages of scanning at 3 T, however, such as increased SAR, increased acoustic noise and more artefacts arising from, for example, susceptibility effects and field homogeneity problems. The problem with artefacts is compounded by the small size of the investigational 3-T neonatal scanner and our clinical readers considered MR artefacts to be a concern in 25% of cases (although all cases were considered to be of diagnostic quality).

The safety of the babies was of paramount importance and required close collaboration between the MR and neonatal unit staff involved in transfer and scanning so that rapid assessment and transfer of babies back to the wards could be made if necessary. This was not required in any of the 52 babies scanned in this study although a consultant-level opinion was sought in three cases (one baby with a bradycardia and two babies with unexpected intra-cranial findings). The increased acoustic noise associated with using a 3-T MR scanner was successfully managed by the use of two types of ear protection. One of the major factors that influences image quality in neonatal imaging is movement of the baby and we have taken a feed and swaddle approach, which appears highly beneficial for settling the babies. Only 1 of 52 of the scans was stopped early because of subject movement and that examination was considered to be partially evaluable by the assessors. Close physiological monitoring of the babies did not reveal any adverse effects leading to problems with desaturation or thermoregulation. Our results showed no problems with temperature control and support the findings of Cawley et al. who measured the core temperatures of neonates during scanning at 3 T and found no significant effects [13]. We must stress, however, that the babies included in this study were “physiologically stable”, which may explain the absence of serious adverse events during the scan procedure. Similar studies are required in the future when acutely unwell and particularly low-birth-weight premature babies will be scanned.

At the planning stage of this study we were prepared to take image quality that was ‘no worse’ than that obtained from our routine clinical scanning on a 1.5-T system as an acceptable outcome. This ‘equivalence’ approach was justified by the extra advantages in terms of safety by having the investigational 3-T neonatal scanner on the neonatal unit. Our results show that the image quality was at least equivalent to 1.5 T in the majority of cases (90%) and were judged to be better than the 1.5-T images in 65% of cases. The investigational 3-T neonatal scanner has the full range of MR sequences that we would expect to use in the clinical environment, all of which produce high-quality images. The next stage of the programme is to see if this can be reproduced in babies that have acute brain pathology. Once CE marking is obtained we will start to perform standard-of-care neuroimaging on that scanner. We will judge whether the optimised sequences developed through the study demonstrate the pathology or if further scan parameter optimisations are necessary.

In conclusion, we describe a new class of high-field MR scanner designed specifically for imaging neonates that is small enough to be sited on neonatal units. Our initial assessments of safety and technical performance are favourable and the next stage is to move on to evaluate diagnostic performance and diagnostic/clinical impact.

## Abbreviations

cUSS: Cranial ultrasonography
CE: European conformity
MHRA: Medicines and Healthcare Products Regulatory Agency of the United Kingdom
MR: Magnetic resonance
SAR: Specific absorption rate
US FDA: United States Food and Drug Administration
